# Ricolinostat, a selective HDAC6 inhibitor, shows anti-lymphoma cell activity alone and in combination with bendamustine

**DOI:** 10.1007/s10495-017-1364-4

**Published:** 2017-03-17

**Authors:** Maria Cosenza, Monica Civallero, Luigi Marcheselli, Stefano Sacchi, Samantha Pozzi

**Affiliations:** Program of Innovative Therapies in Oncology and Haematology, Department of Diagnostic Clinical and Public Health Medicine, University of Modena and Reggio Emilia, Via del Pozzo, 71, 41124 Modena, Italy

**Keywords:** Lymphoma, Ricolinostat, Bendamustine, Apoptosis, Synergistic effect

## Abstract

Histone deacetylase inhibitors (HDACis) have emerged as a new class of anticancer agents, targeting the biological process including cell cycle and apoptosis. We investigated and explained the anticancer effects of an HDAC6 inhibitor, ricolinostat alone and in combination with bendamustine in lymphoma cell lines. Cell viability was measured by MTT assay. Apoptosis, reactive oxygen species (ROS) generation, Bcl-2 protein expression, cell cycle progression and tubuline expression were determined by flow cytometry. The effects of ricolinostat alone and in combination on the caspases, PI3K/Akt, Bcl-2 pathways, ER stress and UPR were assessed by immunoblotting. Ricolinostat shows anti lymphoma activity when used as single agent and its capability to induce apoptosis is synergistically potentiated by the bendamustine in lymphoma cell lines. Drug combination reduced the proportion of cells in the G_0_/G_1_ and S phases and caused an increase of “sub-G_0_/G_1_” peak. The synergistic effect accompanied with the increased ROS, activation of caspase-8, -9, and -3, the cleavage of PARP and modulated by Bcl-2 proteins family. In addition, the exposure of ricolinostat induced the acetylation level of α-tubulin, the extend of which was not further modified by bendamustine. Finally, the apoptosis effect of ricolinostat/bendamustine may be mediated by a corresponding effect on microtubule stabilization. Our data suggest that ricolinostat in combination with bendamustine may be a novel combination with potential for use as an antitumor agent in lymphoma.

**Electronic supplementary material:**

The online version of this article (doi:10.1007/s10495-017-1364-4) contains supplementary material, which is available to authorized users.

## Introduction

Many cellular functions, including cell cycle arrest and apoptosis, are regulated by histone and non-histone proteins that are controlled by protein acetylation. The acetylation state of proteins is controlled by two opposing enzyme classes: histone acetyltransferases (HATs) and histone deacetylases (HDACs) [1]. HDACs regulate gene expression and enzymatically remove the acetyl group from histones [2]. In some disease models including T–cell lymphoma (TCL) and Hodgkin lymphoma alterations were found in histone acetylation; this is correlated with an aggressive disease course and poor treatment outcomes [3, 4]. HDAC inhibitors (HDACi) are a novel class of drugs involved in the modification of epigenetic regulation that are being evaluated in clinical trials in hematological malignancies alone and in combination with approved drugs and with a good safety profile [5–7]. HDACi target tumor cells changing the acetylation of chromatin-associated histones [8], as well as a range of non-histone proteins, with diverse and important biological functions, including transcription factors involved in regulation of cell proliferation, migration and cell death [9, 10]. HDACi can either be pan inhibitors that broadly target different HDAC enzymes, or selective inhibitors that target specific isozymes of HDAC [11]. The development of isozyme selective drugs may provide a therapeutic benefit by minimizing toxicity. HDAC6 is responsible for tubulin deacetylation which plays a key regulatory role in the dynamic stability of the microtubules [12]. In hematological malignancies, HDAC6 has been reported to be overexpressed in primary and cultured multiple myeloma cells and T-cell lymphoma [13, 14].

Ricolinostat (ACY-1215) inhibits HDAC6, resulting in tubulin hyperacetylation [15] and interacts synergistically in combination with bortezomib and carfilzomib to treat lymphoma and multiple myeloma (MM) cells [16–18]. Preclinical studies also confirmed the synergistic effects of HDACi in combination with conventional alkylating agents [19]. It may be a strategy to use an HDAC6 inhibitor in combination with a broad-spectrum chemotherapeutic agent in the clinic to reduce the possibility of developing resistance. A recent study showed that HDACi synergistically enhanced the anticancer effect of bendamustine in multiple myeloma cells [20]. Bendamustine is a bifunctional compound which possess the activity of alkylating and purine analogue agents and demonstrated important results with a good toxicity profile in the therapy of indolent lymphomas, chronic lymphocytic leukemia (CLL), MM, and mantle cell lymphomas (MCL) [21–23]. Bendamustine activates apoptosis pathways causing mitotic catastrophe [24].

The aim of this in vitro study was to investigate the activity of ricolinostat alone and in combination with bendamustine to affect cell viability and apoptotic pathways in a panel of non-Hodgkin’s lymphoma (NHL) cell lines. Our results suggest that combination treatment produced a strong cytotoxic effect when incubated with lymphoma cell lines at concentrations that do not affect normal cell viability. Demonstration of such cytotoxic effects coupled with the improved safety profile of a selective HDAC6 inhibitor [25], provide the rationale for the development of this combination in the treatment of patients with lymphoma.

## Materials and methods

### Reagents and cells culture

Ricolinostat (ACY-1215) and ACY-241 were kindly provided by Acetylon Pharmaceuticals (Boston, USA). ACY-241 is structurally related to ACY-1215 and selectively inhibits HDAC6 with similar biological effects.

Bendamustine was purchased from Selleck Chemicals. Reagents were dissolved in DMSO (Sigma Aldrich), and stored at −20 °C until use. In all experiments, the final concentration of DMSO which was used as vehicle control did not exceed 0.01%. Ricolinostat was investigated using a panel of six NHL cell lines: WSU-NHL, RL (follicular lymphoma, FL), Granta-519, Jeko-1 (mantle cell lymphoma, MCL), Hut-78 (cutaneous T cell lymphoma, CTCL) and Karpas-299 (anaplastic large cell lymphoma, ALCL). WSU-NHL, RL, Granta-519, Jeko-1 and Karpas-299 were purchased from the German Collection of Microorganisms and Cell Cultures (DSMZ). Hut-78 was purchased from the European Collection of Cell Cultures (ECACC). With the exception of GRANTA-519, lymphoma cell lines were cultured in RPMI-1640 supplemented with 10% fetal bovine serum (FBS), 2 mM glutamine, and 100 U/mL penicillin and streptomycin. For Granta-519 cells, DMEM was used in place of RPMI-1640. Cell lines used in this study were thawed from early passage stocks and were passaged for less than 6 months.

Bone marrow mesenchymal stromal cells (BM-MSCs) were generated as previously described [26]. Peripheral blood mononuclear cells (PBMCs) were obtained from two patients with FL, two patients with MCL, one patient with CTCL and from three healthy volunteers using the Ficoll–Hypaque technique. The protocol was approved by the local Institutional Review Board. Written informed consent was obtained before the collection of the samples. All reagents were purchased from Euroclone.

### Viability assay and clonogenic formation

Cell viability was evaluated by MTT colorimetric assay (CellTiter non-radioactive cell proliferation assay, Promega Corporation, Madison, USA), following the manufacturer’s instructions. NHL cell lines were incubated in triplicate with increasing concentrations of ricolinostat (0.01–100 µM) and bendamustine (25–300 µM) as single agents for 24–72 h to identify the IC_50_ values of each drug. For assessment of drug combination effect, serial dilutions of the two agents were assessed using concentrations lower than the IC_50_. NHL cell lines were cultured with fixed doses of ricolinostat (2, 2.5, 4, 5, 8, 10 µM) and bendamustine (10, 20, 25, 40, 50, 100 µM).

For clonogenic assays, NHL cell lines were first exposed to ricolinostat alone and in combination with bendamustine in liquid culture for 24–48 h, then collected and incubated in methylcellulose and maintained for 10 or 14 days. Growing colonies (>50 cells) were counted under a microscope.

### Co-culture of lymphoma cell lines with BM-MSCs

BM-MSCs (5 × 10^4^ cells/well) were seeded in triplicate onto 96-wells plates, and incubated for 48 h to reach confluence. After 48 h, lymphoma cell lines were seeded at 2 × 10^4^ cells/well in the presence or absence of BM-MSCs. The next day, cells were treated with ricolinostat alone or in combination with bendamustine. Non-adherent cells were collected at 24 and 48 h after addition of the drugs, and cell viability was evaluated.

### Cell cycle distribution

Cell lines were cultured at 1 × 10^6^ cells/well for 24–48 h with ricolinostat alone and in combination with bendamustine. Cell cycle analysis was determined by flow cytometry as described previously [27].

### Assessment of apoptosis

Apoptosis was quantified using the Annexin V-FITC and propidium iodide (PI) binding assay, following the manufacturer’s instructions (Miltenyi Biotec, Germany), and analyzed by flow cytometry (FACS Calibur, BD) and Cell Quest data analysis software. Apoptotic cells were designated as Annexin V^+^/PI^−^ and Annexin V^+^/PI^+^, showing early and late apoptosis, respectively.

### Analysis of Bcl-2 expression

After treatment, the cells were fixed and permeabilized using the BD Cytofix/Cytoperm Kit™ (BD Biosciences, San Jose, CA, USA) according to the manufacturer’s instructions. Cells were incubated with FITC-conjugated mouse anti-human Bcl-2 monoclonal antibody (BD Biosciences, San Jose, CA, USA), or FITC-conjugated mouse IgG1 monoclonal isotype control antibody (BD Biosciences, San Jose, CA, USA), then analyzed by flow cytometry.

### Assessment of reactive oxygen species generation

Cells treated for 24 h, were incubated with 5 µM of 2′,7′-dichloroflourescein diacetate (DCFH-DA; Sigma-Aldrich St. Louis, MO, USA) in PBS at 37 °C for 30 min. The free radical scavenger acetyl-l-cysteine (NAC) (Sigma-Aldrich St. Louis, MO, USA) to assess the role of ROS generation in apoptosis. Cells were pre-incubated with 12 mM NAC for 3 h followed by incubation with ricolinostat and bendamustine either alone or in combination. H_2_O_2_ was used as a positive control. The fluorescence intensity was read by flow cytometry on the FL1 channel within 45 min. ROS production was determined in gated live cells by comparing the intensity of fluorescence in treated versus untreated cells. The data were analyzed by Cell Quest data analysis software.

### Western blot analysis

Cell pellets were resuspended in cold lysis buffer (Mammalian Cell Extraction Kit; Biovision Inc. CA, USA) following the manufacturer’s instructions. Cell lysates (50–100 μg of protein) were loaded onto pre-cast 4–20% (w/v) Miniprotean TGX Precast Gels (Bio-Rad, USA), subjected to electrophoresis, and electrotransferred onto nitrocellulose membranes (Bio-Rad, USA). The membranes were incubated overnight at 4 °C and were probed with antibodies against the following protein: AKT, p-AKT (Ser473), GSK-3β, p-GSK-3β (Ser9), p70S6, p-p70S6 (Thr421/Ser424), m-TOR, p-m-TOR (Ser2448), p90/RSK, p-p90/RSK (Thr359/Ser363), 4EBP1, p-4EBP1 (Thr37/46), p21, p27, cyclin E, cyclin D, cyclin B, Bip, p-IRE1α, IRE1α, CHOP, p-PERK, PERK, ATF6 (Pierce), Thioredoxin 1 (Tema Ricerca), Bax, Total Bad, p-Bad (Ser112), p-Bad (Ser136), Bim, BCL-xL, Mcl-1, HDAC6, acetyl-alpha tubulin, caspase 8, caspase 3 (Asp175), caspase 9 (Asp353) and PARP. Caspases and PARP expressions were evaluated also after 1 h of pretreatment with 40 µM of zVAD-fmk (Sigma), a broad caspase inhibitor. The majority of antibodies were purchased from Cell Signaling Technology. For protein loading control, the blots were stripped and reprobed with anti-α-tubulin (Sigma) antibody to ensure equal protein loading. Images were acquired and analyzed using Image Lab Software v.3.0 (Chemidoc Imaging System, Bio-Rad).

### Measurement of IL-10

After 24 h, the cell suspension was carefully centrifuged and cell culture supernatants collected for subsequent IL-10 analysis. IL-10 expression was measured using a commercially available IL-10 enzyme-linked immunosorbent assay (ELISA; R&D systems), according to the manufacturer’s instructions.

### Analysis of tubulin expression

Cells were exposed to ricolinostat alone and in combination with bendamustine and, 24 h later, processed for the tubulin polymerization assay. Samples were prepared as described by Morrison KC et al. [28]. For each sample, the mean fluorescence intensity was recorded using a FACS Calibur cytometer and analyzed using Cell Quest Software. Tubulin levels were determined based on the geometric mean of the antibody/FITC fluorescence and were normalized to a value of 100 for the vehicle control. Paclitaxel and nocodazole were used as positive and negative controls. Paclitaxel would induce the microtubule polymerization; in contrast, nocodazole induces depolymerization of microtubules. All reagents were purchased from Sigma Aldrich.

### Statistical analysis, isobologram and combination index calculation

The effectiveness of the drugs and their combinations used in the present study were analysed using Calcusyn Software. The combination index (CI) and isobologram plot were calculated according to the Chou–Talalay method [29]. Synergism, additivity, or antagonism were quantified by determining the combination index (CI) calculated by the Chou–Talalay equation. We assumed that CI < 1, CI = 1, and CI > 1 indicate synergistic, additive, and antagonistic effects, respectively. All in vitro experiments were performed in triplicate, and repeated at least three times; a representative experiment was selected for the figures. Data are expressed as mean value ± standard error.

Statistical differences between controls and drug-treated cells were determined by one-way analysis of variance (ANOVA). *p* values < 0.05 were considered statistically significant. Data were analysed using the Stata 8.2/SE package (StataCorp LP).

## Results

### Ricolinostat has a cytotoxic effect in lymphoma cell lines

**Fig. 1:**
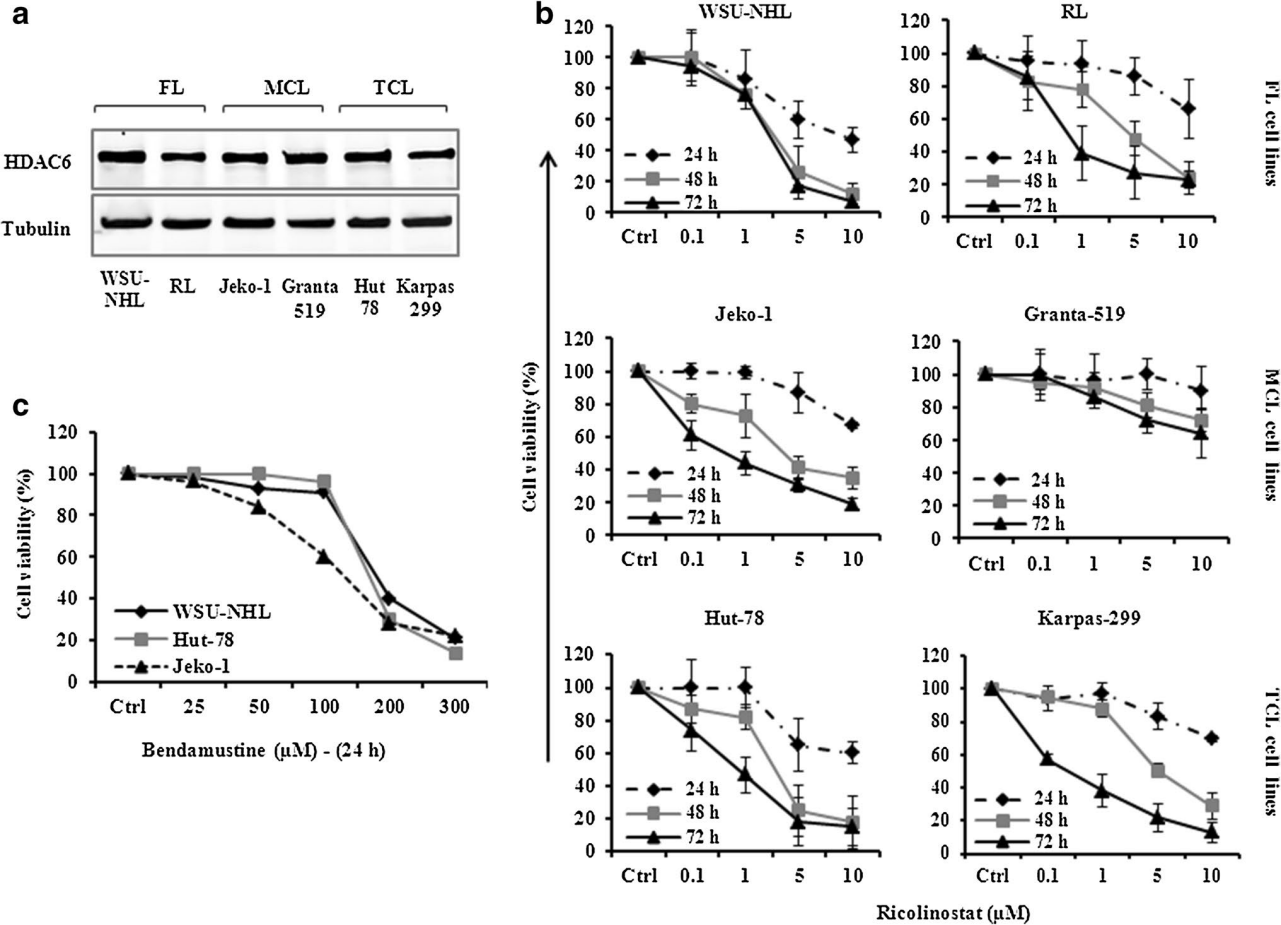
**a** HDAC6 is expressed in six lymphoma cell lines. Whole-cell lysates were subjected to western blotting using the indicated Abs. Tubulin was used to normalize protein loading. **b** Ricolinostat alone induced dose and time dependent manner growth inhibition in NHL cell lines that were treated with a serial dosage of ricolinostat (1–10 µM) for 24–72 h. Data shown are representative of at least three independent experiments and represent the mean ± SD. **c** Antiproliferative activity of bendamustine (25–300 µM) for 24 h. Values represent three independent experiments and represent the mean ± SD

HDAC6 protein was expressed in all six NHL cell lines examined (Fig. 1a). The effect of ricolinostat on lymphoma cell viability was evaluated with escalating concentrations of ricolinostat (0.01–100 µM) for 24–72 h. Exposure to ricolinostat resulted in time and dose-dependent inhibition of cell viability with IC_50_ values ranging from 1.51 to 8.65 μM. Significant cytotoxic effect was observed after 48 h of treatment in five out of six lymphoma cell lines present in the panel. The most sensitive cell lines were WSU-NHL and Hut-78 (IC_50_: 1.97–1.51 μM) and the less sensitive the MCL cell line Granta-519 (IC_50_: 20–64 µM) (Fig. 1b; Supplemental Table S1).

### Growth inhibition of lymphoma cell lines by bendamustine alone

Bendamustine (25–300 μM) induced time and dose-dependent inhibition of cell viability in lymphoma cell lines after 24–48 h with an IC_50_ value after 24 h of 168, 127 and 144 µM for WSU-NHL, Jeko-1 and Hut-78 cells, respectively (Fig. 1c). At 48 h, the IC_50_ value ranged from 83 to 106 µM for the same cell lines (data not shown).

### Drug combination inhibited cell viability in a synergistic manner

**Fig. 2:**
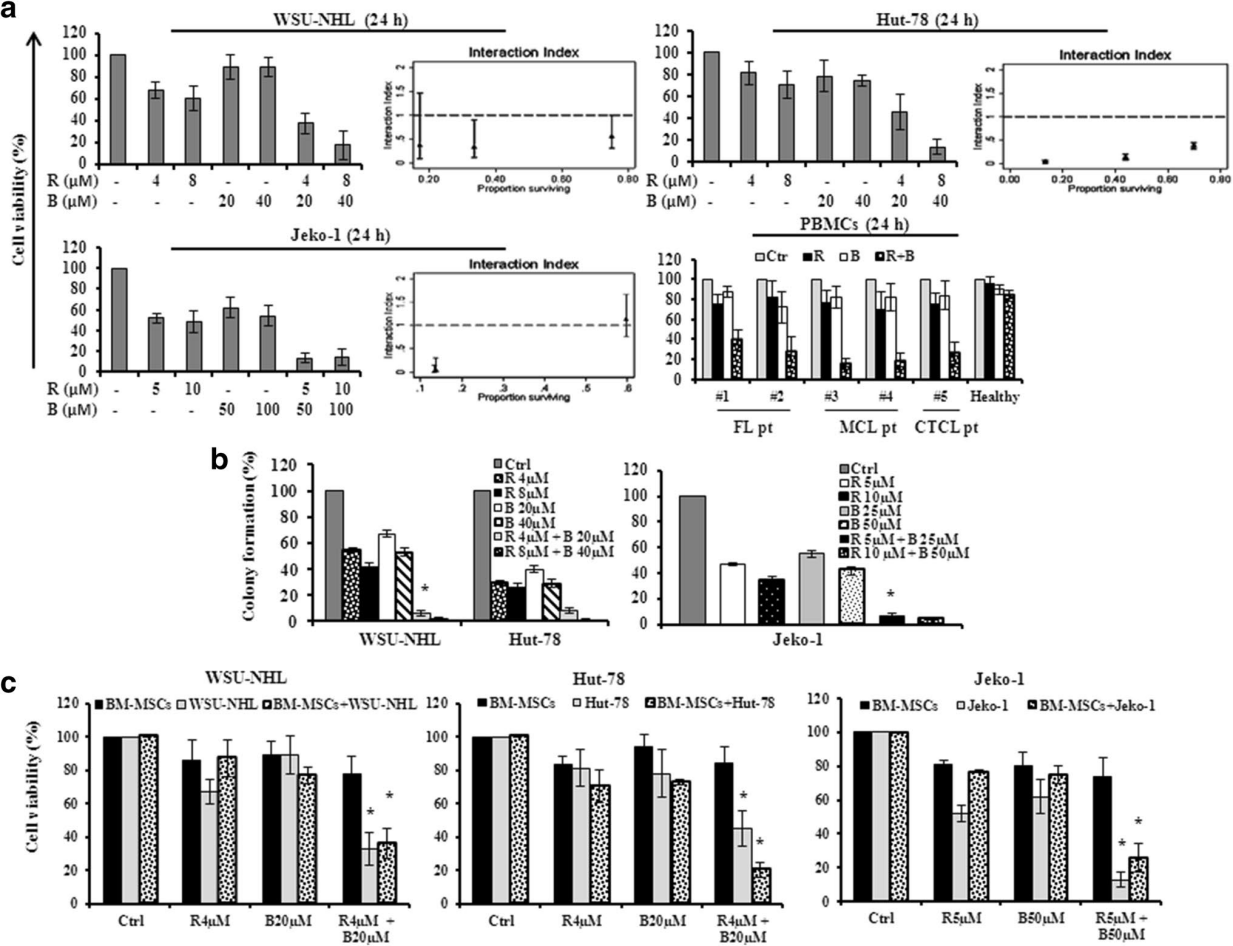
**a** Synergistic effect of drug combination on cell viability of WSU-NHL, Hut-78, Jeko-1, cell lines and PBMCs isolated from two FL patients (Pt#1, Pt#2), two MCL patients (Pt#3 and Pt#4), one CTCL patient and three healthy subjects. The synergistic effect is confirmed with the isobologram analysis (interaction index <1). Data are expressed as a percentage of untreated control cells and represent the mean ± SD of triplicate culture. **b** Effects of drug combination on clonogenic survival. WSU-NHL, Hut-78 and Jeko-1 cells were treated with ricolinostat (R) and bendamustine (B), at the indicated doses, in liquid culture for 24 h. After treatment, cells were incubated in methylcellulose and colonies consisting of more 50 cell were counted after 10 days. The relative percentage with respect to control cells are shown and represent the mean ± SD of three separate experiments (*p < 0.001 vs R and B). **c** Cell viability of WSU-NHL, Hut-78 and Jeko-1 cells co-cultured with or without BM-MSCs and exposed to drugs alone and in combination, at the indicate doses, for 24 h. All data are expressed as a percentage of untreated control ± S.D of triplicate culture (*p < 0.001 vs R and B)

The sensitive lymphoma cell lines of the panel (WSU-NHL, Hut-78 and Jeko-1) were treated with increasing concentrations of ricolinostat (2, 2.5, 4, 5, 8 and 10 μM) in combination with bendamustine (10, 20, 25, 40, 50 and 100 μM) and cell viability was assayed by MTT. The combination studies were performed at 24 h before the start of extensive apoptosis. Even if each drug alone was able to affect the cell viability in a dose dependent manner, the combination drug treatment caused much stronger cytotoxic effect in all cell lines tested. Analysis using the Chou–Talalay method indicated that the effect of the combination was synergistic in all the tested concentrations. A clear synergistic interaction was observed using concentrations lower than the IC_50_ after 24 h of treatment. After 24 h, ricolinostat (2, 4 and 8 μM) and bendamustine (10, 20 and 40 μM) showed a synergistic interaction with a combination index (CI) raging between 0.027 and 0.553 in WSU-NHL and Hut-78 cells, respectively (Fig. 2a; Table 1). The combination of ricolinostat (5, 10 μM) with bendamustine (50, 100 μM) showed a CI of 0.02 and 0.04 in Jeko-1 cells (Fig. 2a; Table 1). Combination treatment also decreased the percentage of viable PBMCs from patients with lymphoma but had minimal or no cytotoxic effect on PBMCs from healthy donors (Fig. 2a). Separate study of sequential treatment with ricolinostat before or after bendamustine enhanced cytotoxicity but was less synergistic than simultaneous treatment (data not shown). Based on the results of the combination in each cell line, we tested the dose of 4 µM of ricolinostat and 20 µM of bendamustine for WSU-NHL and Hut-78 cells and the dose of 5 µM of ricolinostat and 50 µM bendamustine for Jeko-1 cells. At these doses, which are lower than the IC_50_, we reached the CI < 1.

**Table 1:** Analysis of drug combination effects

Ricolinostat (µM)	Bendamustine (µM)	Effect	CI (CI 95%)
WSU-NHL			
2	10	0.75	0.553 (0.307–0.996)
4	20	0.34	0.324 (0.116–0.900)
8	40	0.17	0.305 (0.091–1.46)
Hut-78			
2	10	0.70	0.374 (0.313–0.446)
4	20	0.44	0.124 (0.079–0.193)
8	40	0.13	0.027 (0.011–0.067)
Jeko-1			
2.5	25	59.6	1.13 (0.77–1.67)
5	50	13.3	0.04 (0.01–0.15)
10	100	13.7	0.02 (0.01–0.31)

Lymphoma cell lines were cultured with fixed doses of ricolinostat and bendamustine alone and in combination. Synergism, additivity, or antagonism were quantified by determining the CI calculated by the Chou–Talalay equation. Combination index (CI): CI < 1, synergism; CI = 1, additive effect; CI > 1, antagonis

### Drug combination affects clonogenic survival of NHL cells and overcomes the protective effect of BM-MSCs

We studied the effect of the drug combination on self-renewal by examining clonogenic growth in methylcellulose. Colony formation reflected clonogenic potential at the end of the treatment period in liquid culture (24 h). Clonogenic assay revealed that the drug combination inhibited colony formation significantly compared with the drugs alone (Fig. 2b). We next examined whether ricolinostat/bendamustine inhibited cell viability even in the presence of BM-MSCs. WSU-NHL and Hut-78 cell lines were co-cultured with BM-MSCs and treated with 4 µM of ricolinostat and/or 20 µM of bendamustine, while Jeko-1 was treated with 5 µM of ricolinostat and 50 µM of bendamustine and cell viability was assessed by MTT. Drug combination decreased cell viability of lymphoma cell lines co-cultured with BM-MSCs, indicating that it overcomes the protective effects conferred by the bone marrow microenvironment and the combination had minimal or no cytotoxic effect on BMSCs (Fig. 2c).

### Ricolinostat/bendamustine affected the cell cycle through the regulatory proteins p21 and p27

**Fig. 3:**
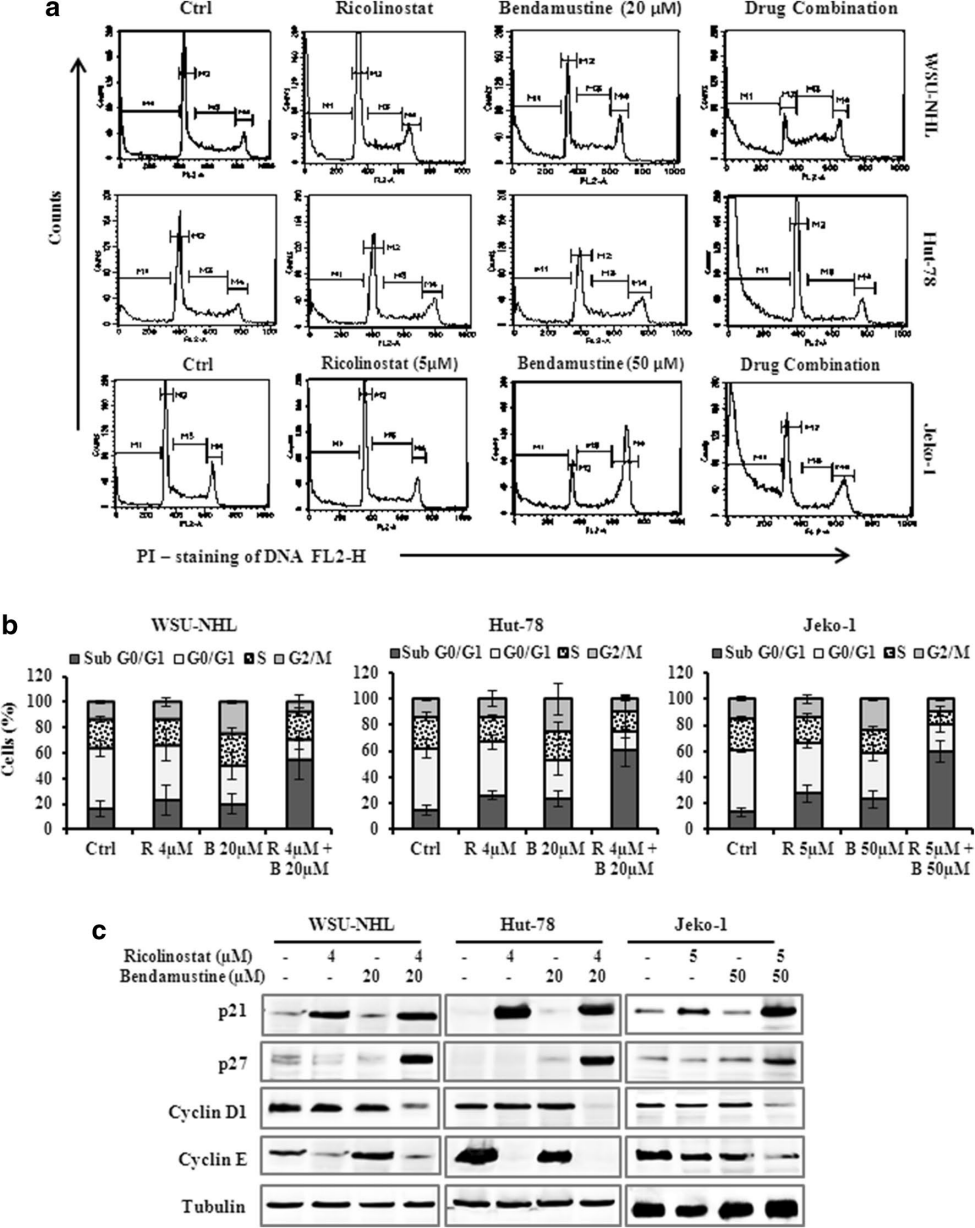
**a** Representative cell cycle profile of WSU-NHL, Hut-78 and Jeko-1 treated at the indicated doses for 24 h. The bars of M1, M2, M3 and M4 indicate the sub-G_0_/G_1_, G_0_/G_1_, S and G_2_/M phases, respectively. **b** cell cycle distribution (%) of lymphoma cell lines in different phases after 24 h of treatment. Values represent the mean ± SD of three independent experiments. **c** WSU-NHL, Hut-78 and Jeko-1 treated with the drugs alone or in combination for 24 h. Whole-cell lysates were subjected to Western blotting using the indicated Abs. Tubulin was used to normalize protein loading

Ricolinostat alone induced an increase of the percentage of cells in the G_0_/G_1_ phase compared with untreated control, while the drug combination reduced the proportion of cells in the G_0_/G_1_ and S phases and caused an increase of “sub-G_0_/G_1_” peak (Fig. 3a, b). To further characterize the cell-cycle regulatory effects of ricolinostat alone and in combination, we analyzed the levels of cell-cycle regulatory proteins, including cyclin D1, cyclin E, p21, p27, which control G_1_/S transition. The treatment with drug combination for 24 h caused a decrease of cyclin D1, and cyclin E in lymphoma cells, in parallel the level of p21 protein and p27 increased (Fig. 3c).

### Apoptosis induced by drug combination is mediated by Bcl-2 family proteins and caspase activation

**Fig. 4:**
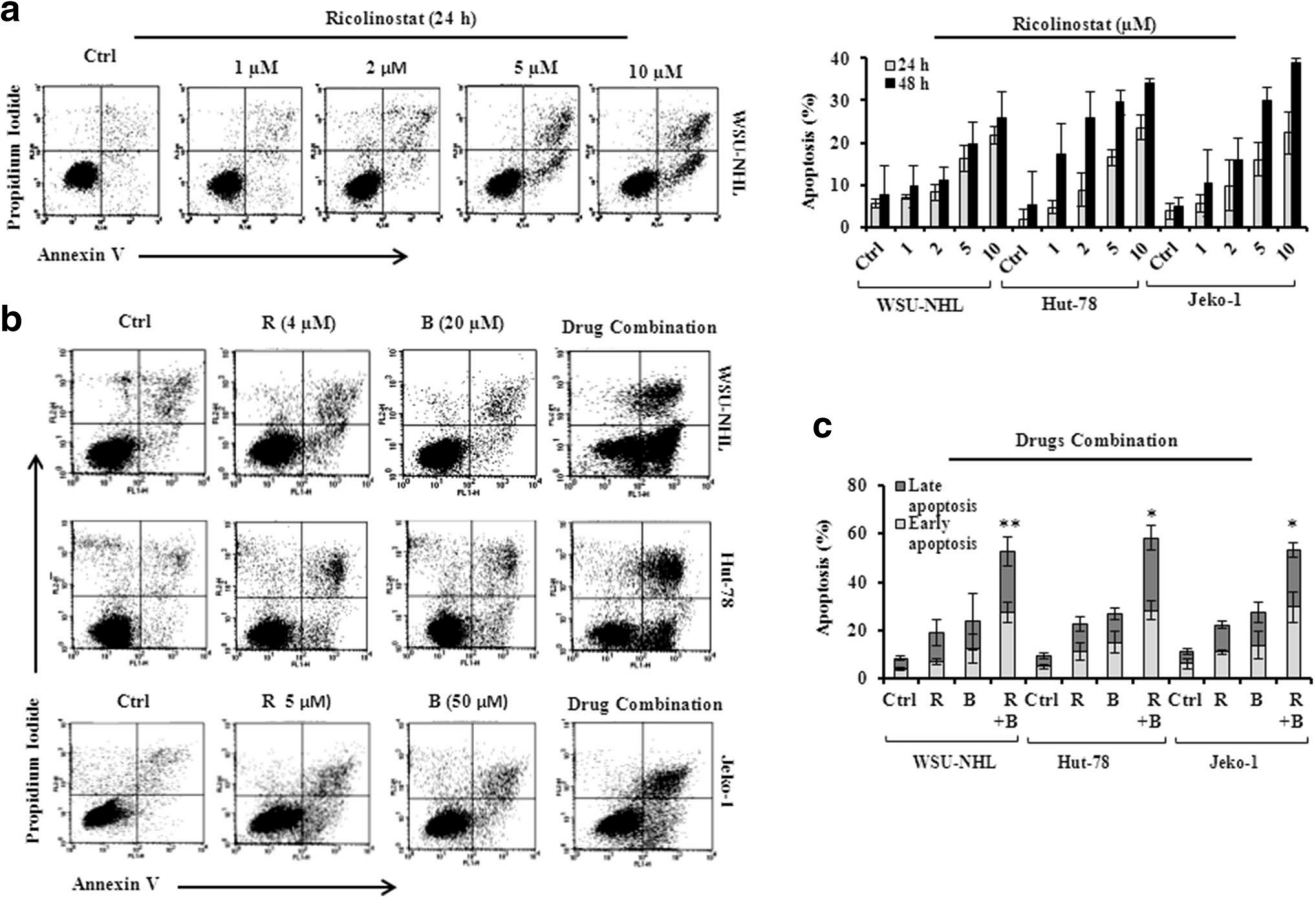
**a** Representative dot blot of WSU-NHL cells treated with ricolinostat alone (1, 2, 5, 10 µM) for 24 h and assayed for apoptosis by annexin V/PI staining (*left panel*); Percentages of apoptotic cells of WSU-NHL, Hut-78 and Jeko-1 cell lines after 24–48 h of exposure to ricolinostat alone (*panel right*). Values represent the mean ± SD of three independent experiments. **b** Representative dot blot of WSU-NHL, Hut-78 cells treated with 4 µM of ricolinostat in combination with 20 µM of bendamustine for 24 h and Jeko-1 cells treated with combination of 5 µM ricolinostat and 50 µM bendamustine. The flow cytometry shows an increase of apoptosis induced by combination. **c**, Percentages of apoptotic cells (early and late apoptosis) treated with ricolinostat (R) and bendamustine (B) as above (*p < 0.001 vs Ctrl; **p = 0.003 vs Ctrl)

**Fig. 5:**
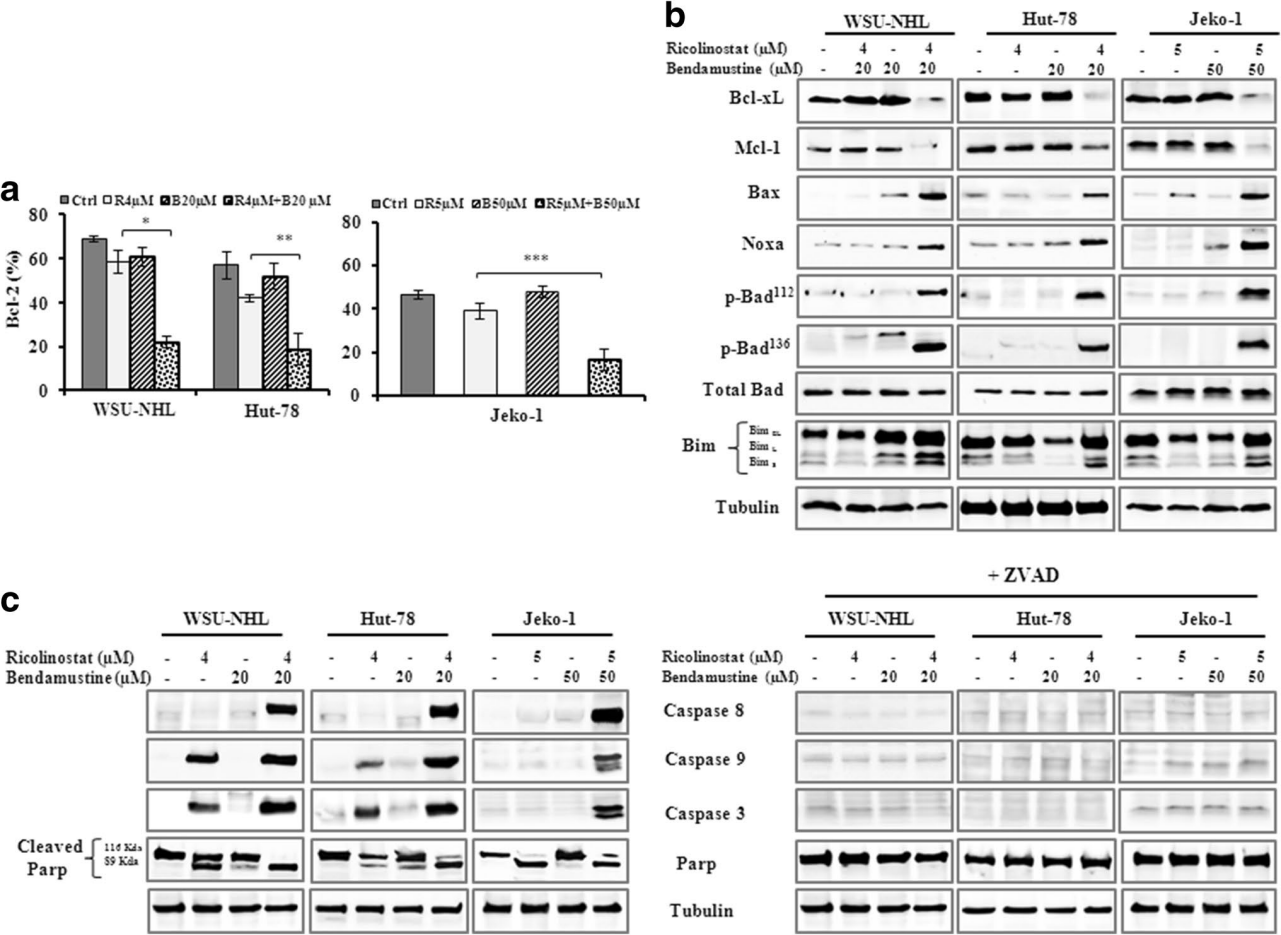
**a** Bar graph shows the representative data (%) of Bcl-2 levels in WSU-NHL, Hut-78 and Jeko-1 cells, evaluated by flow cytometry (*p = 0.001; **p = 0.009; ***p = 0.002). **b** Drug combination mediated the down regulation of anti-apoptotic proteins and phosphorylation of the pro-apoptotic proteins. Whole-cell lysates were subjected to western blotting using the indicated Abs. Tubulin was used to normalize protein loading. **c** Representative western blot for caspases-8, -9, -3 and PARP with or without ZVAD in cellular extracts from WSU-NHL, Hut-78 and Jeko-1 cells. Tubulin is shown as a loading control

Ricolinostat alone induced apoptosis in all cell lines examined in a time and dose dependent manner (Fig. 4a). This effect was enhanced by adding bendamustine to ricolinostat. After 24 h, ricolinostat/bendamustine induced significantly greater apoptosis compared with either drug alone (Fig. 4b, c). Combination treatment at 48 h was too toxic to be assessed (data not shown). Since the involvement of Bcl-2 family proteins in DNA damage-induced apoptosis is well-known, we examined the expression of Bcl-2 family members including anti-apoptotic proteins and pro-apoptotic proteins. In comparison to the effects of the single treatments, the drug combination reduced the protein level of Bcl-2 (Fig. 5a), Bcl-xL and Mcl-1 (Fig. 5b) and increased the levels of the pro-apoptotic members of Bcl-2 family, such as Bax, Bim, Noxa, p-Bad^112^ and p-Bad^136^ (Fig. 5b). Ricolinostat alone and in combination induced PARP cleavage, the hallmark of apoptosis and activation of caspase-8, -9 and -3 in all three cell lines (Fig. 5c). With the presence of ZVAD, a pan caspase inhibitor, the effect of the drug combination on caspases and PARP cleavage was completely inhibited, indicating that drug combination induced apoptosis by activating the caspase pathway (Fig. 5c).

### Drug combination activated ER stress through ROS generation

**Fig. 6:**
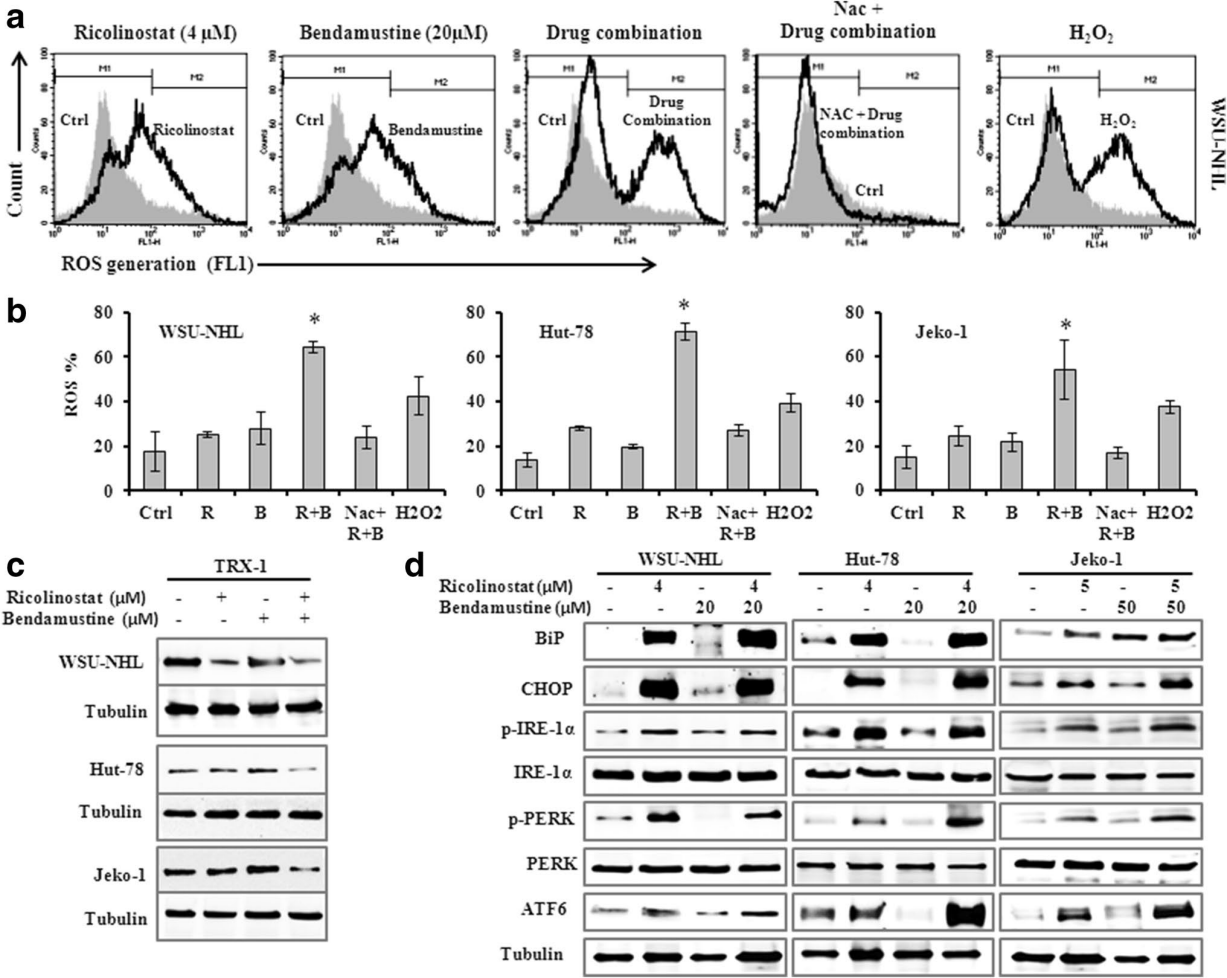
**a** Representative histograms showing ROS level from WSU-NHL after treatment with drugs alone and in combination for 24. The *bar* M2 indicates the fraction of ROS positive cells. **b** Percentage of cells with increased ROS level from drug combination compared with the control cells. The co-administration of the antioxidant NAC blocked the increased of ROS generation. H_2_O_2_ was used as a positive control. Data are expressed as the mean ± SD of triplicate culture. (*p < 0.001 vs ricolinostat and bendamustine). **c** Western blot of cellular extracts from WSU-NHL, Hut-78 and Jeko-1 cells probed with antibody against Trx-1. Tubulin was used to normalize protein loading. **d** Drug combination mediated ER stress and UPR signaling. Representative western blots of cellular extracts from WSU-NHL, Hut-78 and Jeko-1 treated with the drugs alone or in combination at the indicated doses for 24 h. Whole-cell lysates were subjected to western blotting using the indicated Abs. Tubulin was used to normalize protein loading

Since ROS generation is implicated in HDACi mediated cell death [30], we investigated whether ROS might be involved in the synergism between ricolinostat and bendamustine. In WSU-NHL, Hut-78 and Jeko-1, the exposure to ricolinostat alone (1, 5 and 10 µM) resulted in an increase of ROS production: from 10.8 to 27% at 24 h with a further increase at 48 h (from 15 to 36.6%). Ricolinostat/bendamustine in combination induced a significant increase in ROS-positive cells from 54 to 71% in the three cell lines, with fold increase ranging from 2.2 to 3.6 when compared with each drug alone, and co-administration of the antioxidant NAC, a ROS scavenger, reduced the generation of ROS (Fig. 6a, b). ROS generation induced by the drug combination were linked to a decrease of thioredoxin-1 (Trx1) expression (Fig. 6c). The Trx system is an antioxidant system integral to maintaining the intracellular redox state. Trx can also scavenge ROS and directly inhibits pro-apoptotic proteins. The ROS generation and Trx1 inhibition play an important role in the toxicity of combined treatment in lymphoma cell lines. ROS generation is frequently associated with the activation of transcription factors linked with induction of endoplasmic reticulum (ER) stress and this could play a crucial role in apoptosis induced by the drug combination. The accumulation of misfolded proteins in the endoplasmic reticulum causes ER stress and causes the unfolded protein response (UPR) [31]. To evaluate the possible involvement of the ER stress response in apoptosis induced by the drug combination, we analyzed by western blot the possible modification of the protein expression levels of some hallmarks of ER stress such as IRE1-α, ATF6 and PERK and the expression of UPR sensors such as BIP and CHOP. The apoptosis induced by combination treatment correlated with increased expression of IRE1-α, PERK and ATF6, which are three ER stress sensors (Fig. 6d).The UPR stress proteins BiP and CHOP were clearly induced by ricolinostat, and the effect was maintained in the combination with bendamustine.

### Co-exposure to ricolinostat/bendamustine leads to AKT pathway inactivation

**Fig. 7:**
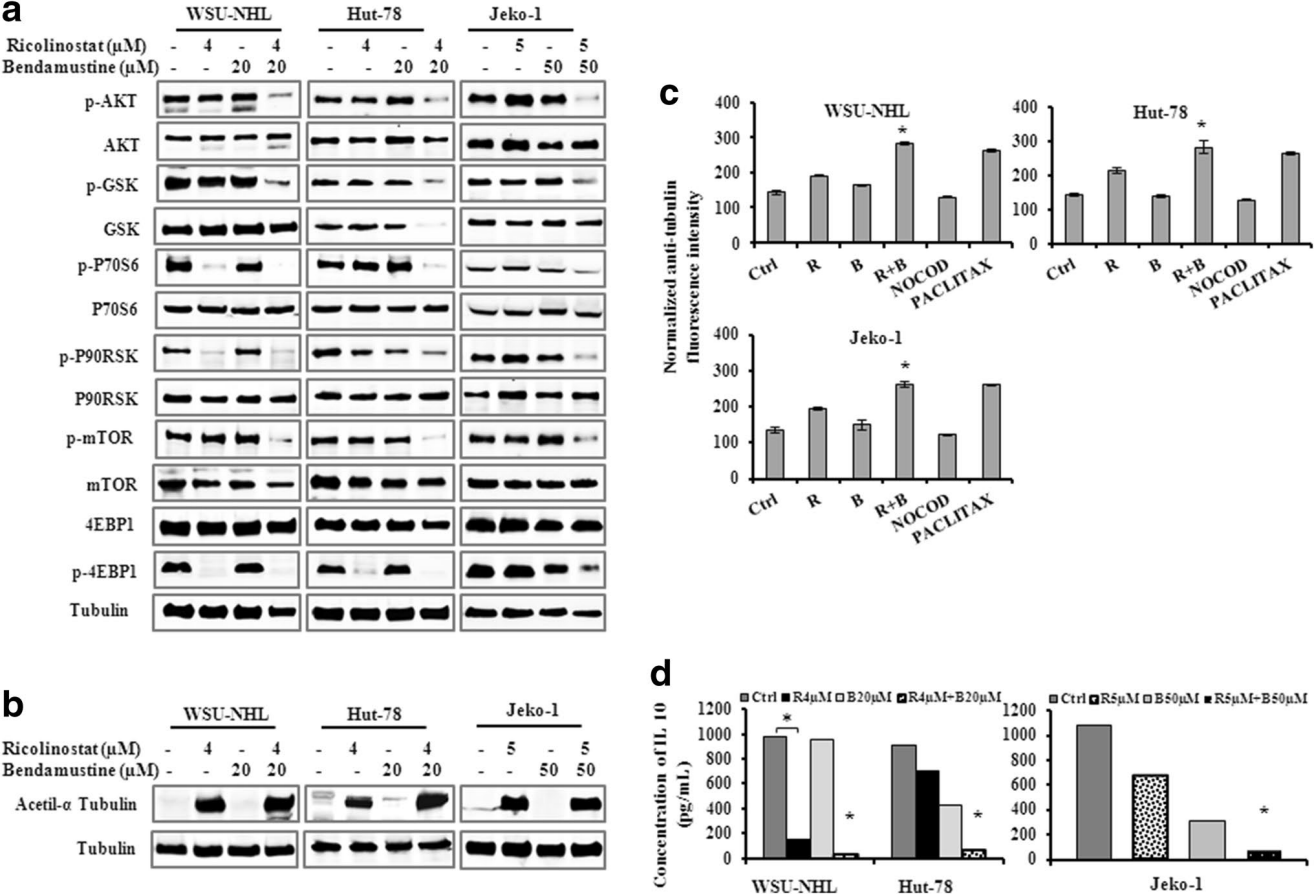
**a, b** Western blots of cellular extracts from WSU-NHL, Hut-78 and Jeko-1 treated with the drugs alone or in combination at the indicated doses for 24 h. Whole-cell lysates were subjected to western blotting using the indicated Abs. Tubulin was used to normalize protein loading. **c** Representative data from analyses of tubulin polymerization assessed by anti-α-tubulin staining and flow cytometry. WSU-NHL, Hut-78 and Jeko-1 treated with ricolinostat alone and in combination at the indicated doses as above for 24 h as well as with the microtubule destabilizer nocodazole (1 μM) and the microtubule stabilizer paclitaxel (100 nM). Data are expressed as mean ± SD and were obtained from three independent experiments performed in triplicate (*p < 0.001 vs R and B alone). **d** Effect of drug combination on IL-10 secretion in WSU-NHL, Hut-78 and Jeko-1 cells treated as above. IL-10 secretion was analyzed by ELISA. Data are means (±SD) of at least three separate experiments each performed in duplicate (*p < 0.001 vs R and B)

AKT pathway is the most important and intensively investigated signaling pathway that plays central roles in governing the cell survival and its dysregulation is related to the development of many diseases. To evaluate the effects of ricolinostat/bendamustine on AKT pathway signaling, we analyzed the phosphorylation status of Akt and some downstream targets including GSK3β, mTOR, 4EBP1, p90RSK and p70S6kinase. Combined treatment induced down-regulation of p-AKT and multiple downstream targets (Fig. 7a).

### Effect of ricolinostat alone and in combination on the acetylation of α-tubulin

Studies showed that ricolinostat increased the acetylation of α-tubulin, a specific substrate of HDAC6 [15]. Using antibodies specifically recognizing acetylated α-tubulin, western blot analysis revealed that exposure of ricolinostat induced the acetylation of α-tubulin in lymphoma cells, the extent of which was not further modified by bendamustine (Fig. 7b).

### Ricolinostat alone and in combination stabilizes microtubules

α-tubulin is a non-histone substrate for HDAC6 enzymes [32] and its overexpression promotes chemotactic cell movement, a function related to the microtubules. The accumulation of acetylated α-tubulin is associated with stabilized microtubule structures, which disrupt the alignment of chromosomes during mitosis and lead to apoptosis. Tubulin deacetylation is associated with microtubule depolymerization, and accumulation of acetylated tubulin following treatment with HDAC6 inhibitors would be expected to lead to microtubule stabilization [28]. To elucidate the effect of ricolinostat alone and in combination with bendamustine on tubulin polymerization, we utilized a cytometric based technique [28] that allows direct quantitative evaluation of tubulin without interference from microtubule-associated proteins or other complicating factors thus enabling facile comparison of compounds that affect tubulin polymerization.

Lymphoma cell lines were treated for 24 h with ricolinostat alone and in combination with bendamustine with either the microtubule destabilizer nocodazole (1 μM) or the microtubule stabilizer paclitaxel (100 nM). This was followed by whole cell-based quantitative measurement of tubulin polymerization using α-tubulin staining. Ricolinostat in combination with bendamustine induced an increase of intensity of fluorescence and acted as a microtubule stabilizer, with an effect similar to paclitaxel Fig. 7c. Nocodazole had the opposite effect, as treatment with this tubulin destabilizer clearly decreased tubulin polymerization.

### Ricolinostat alone and in combination down-modulated IL 10 expression

HDAC6 has been shown to be involved in regulation of inflammatory and immune responses [33]. IL-10 is a multifunctional cytokine produced by diverse immune cell types, including B cells and subsets of T cells. IL-10 has a potent stimulating effect, inducing proliferation and differentiation [34]. Therefore, we studied the expression of IL-10 in lymphoma cell lines after treatment with ricolinostat alone and in combination. Ricolinostat alone induced a significant down-regulation of IL-10, that was especially evident in WSU-NHL with a fold decrease of 6.6 compared to control. The drug combination affected the IL-10 production in all the three cell lines with a fold decrease of 5.77 in WSU-NL; 11.5 in Hut-78; 10.9 in Jeko-1 cells compared with ricolinostat alone (Fig. 7d).

## Discussion

HDACi have emerged as a new class of target therapy and have showed synergy with a number of anticancer drugs. HDACi are known for their selective cytotoxicity that discriminates between normal and tumor cells. Selective inhibition may improve the efficacy and reduce the toxicity of pan-HDAC inhibitors observed in the clinic. HDAC6, a class IIb HDAC, is a key regulator of many signaling pathways that are associated to cancer, thereby making HDAC6 an attractive target. Ricolinostat is a selective HDAC6 inhibitor, which induces synergistic cell cytotoxicity in combination with proteasome inhibitors [15–17] and immunomodulatory agents [35, 36] in MM cell lines and animal models. Ricolinostat has demonstrated an excellent safety and tolerability profile in Phase I trials as a single agent and in drug combinations [36]. Although the antitumor activity of HDACi was confirmed in various studies, it is widely accepted that HDACi in combination with other antitumor drugs may be more effective than HDACi alone. The combination of two compounds with different mechanism of action can lead to a potential synergistic effect and improved pharmacological potency. Preclinical and Phase 1^a^ clinical data [25] support the hypothesis that the safety profile of a selective HDACi will facilitate combination treatment with other active agents such as bendamustine, which has being given new perspective in treating hematologic malignancies such as CLL, lymphoma and MM [37, 38]. Bendamustine is known to cause intra- and inter-strand DNA cross-links that initiate a DNA damage response [24]. Repair of this damage leads to survival of cells; therefore, a strategy to overcome this survival mechanism may be the combination of bendamustine with a drug involved in the modification of epigenetic regulation. We present data indicating that ricolinostat shows anti lymphoma activity as single agent and its ability to induce apoptosis is synergistically increased by bendamustine in lymphoma cell lines. Six cell lines of different histology have been tested. Ricolinostat enhanced bendamustine induced inhibition of cell viability, reduced clonogenic survival and overcame the proliferative advantage of BMSCs with minor toxicity against PBMCs. Drug combination reduced the proportion of cells in the G_0_/G_1_ and S phases and caused an increase of “sub-G_0_/G_1_” peak, modulated Bcl-2 protein family members and activated caspase-3 leading to PARP degradation. Caspase activation occurs through various pathways, such as mitochondria, death receptor and ER pathway. HDACi are known to activate caspases by mitochondrial or death receptor-mediated pathways [39]. There are different studies showing that HDACi induce ROS production and caspase activation [30] including ricolinostat [15]. ROS production, induced by HDACi, leads to activation of caspase and generates apoptosis in various types of cancer cells through an extrinsic or intrinsic pathway [30]. In most cell types the main source of ROS are the mitochondria and anomalies in ROS generation may play an important role in signaling mechanisms of apoptosis. Irregular ROS production can also promote the conformational changes of members of the pro-apoptotic Bcl-2 family and their intervention in increasing the permeability of the mitochondrial membrane. Ricolinostat/bendamustine induced apoptosis via ROS generation and apoptosis was attenuated by pre-incubation with NAC, suggesting that ROS production is likely involved in the mode of action of this drug combination in lymphoma cell lines. ROS production induced by the drug combination was associated with decreased expression of Trx, a ubiquitous protein with pleiotropic effects that functions as an intracellular antioxidant. Trx stimulates tumor growth and inhibits both spontaneous and drug-induced apoptosis [40]. Studies have shown that this antioxidant is upregulated in certain types of tumors [41, 42] possibly giving tumor cells a survival advantage in order to survive to elevated oxidative stress. The overexpression of Trx is associated with resistance to many anticancer drugs and the inhibition of Trx expression may overcome drug resistance and probably sensitize lymphoma cells to other chemotherapeutic agents. Thus, decreasing Trx levels may contribute in the treatment of lymphoma. The mechanism involved in the HDACi induced cell death is still unclear, although, oxidative stress has been identified as a mechanism involved in the cytotoxicity of HDACi but the manner by which HDACi induce oxidative stress is poorly understood. Our results suggest an association between cytotoxicity of the drug combination and ER-stress loading. The expression of ER-stress related proteins demonstrated that treatment with ricolinostat plus bendamustine was a potent combination for ER-stress loading, compared with each drug alone. Bip, which plays a central regulatory role in the unfolded protein response (UPR), represents a protein recently identified, which acetylation is induced by HDAC inhibition, leading to UPR activation. HDAC6, which primarily resides in the cytoplasm, has also been implicated as the primary enzyme contributing to the acetylation of the ER-localized chaperone protein Bip [43]. Specifically, the UPR is a dynamic response to ER stress that may initially serve a protective function but which may ultimately promote cell death [44]. Activated BiP binds the accumulated unfolded proteins and dissociates from ER stress sensors PERK, ATF6, and Ire-1α, inducing ER stress [31]. Histone acetylation is known to result in the opening of condensed chromatin, which is in turn associated with transcriptional activation. A variety of non-histone proteins are subject to acetylation and deacetylation modifications. One non histone target for HDAC6 is tubulin [45]. It has been documented that disturbances in either microtubule assembly or disassembly have destructive effects on cellular functions, ultimately leading to cell death. Our data showed that the drug combination has effects on tubulin acetylation and cell apoptosis. We observed an increase in tubulin acetylation, suggesting that the anticancer activity of ricolinostat/bendamustine may be attributed in part to effects on microtubule stabilization. Acetylation of α-tubulin was not further modified by bendamustine. Nocodazole interferes with the dynamic assembly of microtubule by preventing tubulin polymerization [46]. In contrast, paclitaxel exerts its anticancer effect partly by blocking tubulin depolymerization and consequently stabilizing microtubule [47]. The inhibition of HDAC6 function leads to acetylation of tubulin and microtubules and thus stabilizes microtubule [48]. The fact that these agents interrupt the microtubule dynamics supports the rationale for a combination of these anticancer agents, a strategy that has been explored in preclinical and clinical studies. In conclusion, several studies have demonstrated that HDACi induce oxidative stress in different types of cancer cells and thus can be used as a strategy to treat cancer. Understanding how HDACi can alter the redox status in cancer cells is of critical importance for their development and better design of clinical trials that include combination of HDACi with other anticancer agents. The basis for combination therapy is to combine drugs acting on different mechanisms, thereby potentiating efficacy and decreasing drug resistance that cancer cells may develop.

Our study demonstrated that the HDAC6 inhibitor ricolinostat is effective in reducing lymphoma cell growth and increasing apoptosis as a single agent and the efficacy is increased by the combination with bendamustine. Ricolinostat synergistically enhances bendamustine-induced growth inhibition in lymphoma cells mainly through multiple mechanisms, including ROS generation, ER stress, acetylation of tubulin and induction of apoptosis. These preclinical studies suggest that bendamustine in combination with epigenetic therapy, such as ricolinostat, may be promising treatment regime for managing lymphoma.

## Electronic supplementary material

Below is the link to the electronic supplementary material.

Supplementary material 1 (DOCX 16 KB)
