# Supplementary material for: Ricolinostat, a selective HDAC6 inhibitor, shows anti-lymphoma cell activity alone and in combination with bendamustine
Source: Apoptosis. 2017 Mar 17;22(6):827–40. doi: 10.1007/s10495-017-1364-4 (PMC5401712; doi:10.1007/s10495-017-1364-4)
Supplement: Supplementary file 1 — Supplementary material 1 (DOCX 16 KB) [file 10495_2017_1364_MOESM1_ESM.docx]

**Supplemental Table S1.**

**IC_50_ values for Ricolinostat in lymphoma cell lines.** WSU-NHL, Hut-78 and Jeko-1 cells were treated with ricolinostat at a range of concentrations from 1 to 100 µM for 24h, 48h and 72 hours. IC_50_ values were calculated using MTT assay. CI95%: Confidence Interval. Values represent three independent experiments.

| **Lymphoma cell lines** |  | **24 h** | **48 h** |
| --- | --- | --- | --- |
| **Follicular cell lymphoma** | **WSU-NHL**  IC_50_  CI95% | 8.65  3.01;14.3 | 1.97  1.01;4.95 |
|  | **RL**  IC_50_  CI95% | -  - | 3.37  7.81;14.6 |
| **Mantle cell lymphoma** | **Jeko-1**  IC_50_  CI95% | -  - | 3.42  1,64;8.48 |
|  | **Granta-519**  IC_50_  CI95% | 64  52;76 | 20  13;26 |
| **T - cell lymphoma** | **Hut-78**  IC_50_  CI95% | -  - | 1.51  3.84;6.86 |
|  | **Karpas-299**  IC_50_  CI95% | -  - | 4.82  0.15;9.80 |
